# Primary Hyperparathyroidism and Psychiatry: Manifestations, Causes and Management

**DOI:** 10.1192/bjo.2025.10231

**Published:** 2025-06-20

**Authors:** Sukhmeet Singh, Victoria Stokes, Al Hakam El Kaubaisy, Judy Rubinsztein

**Affiliations:** 1 NHS Greater Glasgow and Clyde, Glasgow, United Kingdom; 2 University of Glasgow, Glasgow, United Kingdom; 3 Addenbrooke’s Hospital, Cambridge, United Kingdom; 4 Cambridgeshire and Peterborough NHS Foundation Trust, Cambridge, United Kingdom; 5 University of Cambridge, Cambridge, United Kingdom

## Abstract

**Aims:** Primary hyperparathyroidism (PHPT) is characterized by hypercalcaemia with an elevated or inappropriately normal parathyroid hormone level. In clinical psychiatry, it is often detected through routine blood investigations for hypercalcaemia. PHPT is the most common cause of persistent hypercalcaemia and the third most prevalent endocrine disorder after diabetes and thyroid disease. Although it can occur at any age, it typically presents later in life, with a mean age of diagnosis of 65 years. Women are affected three to four times more frequently than men. As awareness of PHPT screening increases, the number of individuals receiving mental health treatment who also have PHPT is likely to rise.

This literature review was prompted by the presentation of several patients with primary hyperparathyroidism (PHPT) or lithium-associated hyperparathyroidism (LAH) in our clinical practice. Our aim was to synthesize the available evidence to provide an updated overview for clinicians regarding the relationship between PHPT and both depression and cognitive impairment, as well as to review the existing literature on LAH.

**Methods:** We conducted a narrative review of the literature, focusing on the relationship between PHPT and both depression and cognitive impairment. We did not explore the association between PHPT and psychosis due to the limited literature in this area, which is primarily confined to case reports. Our search yielded 42 full-text articles, which were reviewed in detail by all authors for inclusion in this study. We discussed the findings and reached a consensus. Additionally, we developed three fictionalized case reports based on amalgamated patient presentations from our clinical experience.

**Results:** Moderate to severe depressive symptoms occur in approximately one-quarter of individuals with PHPT before parathyroidectomy, and about two-thirds show improvement one year post-surgery. Further research is needed to guide antidepressant treatment, particularly regarding when to withdraw this post-operatively if the depression may be attributed to PHPT.

**Conclusion:** Patients diagnosed with PHPT should be referred to endocrinology clinics. Current surgical criteria primarily focus on classical PHPT symptoms, such as osteoporosis. However, expert panels recognize that non-specific symptoms, including depression, often improve following parathyroidectomy. This suggests that surgery may be a cost-effective intervention to prevent further health deterioration.

